# Kidney Recovery From Acute Kidney Injury After Hematopoietic Stem Cell Transplant: A Systematic Review and Meta-Analysis

**DOI:** 10.7759/cureus.12418

**Published:** 2021-01-01

**Authors:** Swetha Rani Kanduri, Karthik Kovvuru, Wisit Cheungpasitporn, Charat Thongprayoon, Tarun Bathini, Vishnu Garla, Pradeep Vailta, Saraschandra Vallabhajosyula, Juan Medaura, Kianoush Kashani

**Affiliations:** 1 Nephrology, Ochsner Medical Center, New Orleans, USA; 2 Nephrology and Hypertension, Mayo Clinic, Rochester, USA; 3 Internal Medicine, University of Arizona, Tucson, USA; 4 Internal Medicine, University of Mississippi Medical Center, Jackson, USA; 5 Nephrology, University of Mississippi Medical Center, Jackson, USA; 6 Cardiovascular Medicine, Mayo Clinic, Rochester, USA; 7 Pulmonary and Critical Care Medicine, Nephrology and Hypertension, Mayo Clinic, Rochester, USA

**Keywords:** aki, incidence, recovery, bone marrow transplantation, hematopoietic stem cell transplant, meta-analysis, acute kidney injury

## Abstract

Patients with the recovery of kidney function after an episode of acute kidney injury (AKI) have better outcomes compared to those without recovery. The current systematic review is conducted to assess the rates of kidney function recovery among patients with AKI or severe AKI requiring kidney replacement therapy (KRT) within 100 days after hematopoietic stem cell transplant (HSCT).

Methods

The Ovid MEDLINE, EMBASE, and Cochrane databases were systemically searched from database inceptions through August 2019 to identify studies reporting the rates of recovery from AKI after HSCT. The random-effects and generic inverse variance methods of DerSimonian-Laird were used to combine the effect estimates obtained from individual studies.

Results

A total of 458 patients from eight cohort studies with AKI after HSCT were identified. Overall, the pooled estimated rates of AKI recovery among patients with AKI and severe AKI requiring KRT within 100 days were 58% (95%CI: 37%-78%) and 10% (95%CI: 2%-4%), respectively. Among patients with AKI recovery, the pooled estimated rates of complete and partial AKI recovery were 60% (95%CI: 39%-78%) and 29% (95%CI: 10%-61%), respectively. There was no clear correlation between study year and the rate of AKI recovery (p=0.26).

Conclusion

The rate of recovery from AKI after HSCT depends on the severity of AKI. While recovery is common, complete recovery is reported in about two-thirds of all AKI patients. The rate of recovery among those with AKI requiring renal replacement therapy (RRT) is substantially lower.

## Introduction

Knowledge of acute kidney injury (AKI) has exponentially increased recently. Particularly, the role of AKI on short- and long-term outcomes has been highlighted. AKI is not an isolated benign increase in serum creatinine levels because they predispose individuals to recurrent AKIs, chronic kidney disease (CKD), increased hospitalizations, and end-stage kidney disease [1]. AKI has emerged as a global burden with subsequent risk of cardiovascular disease, stroke, essential hypertension, bone disease, and worse overall long-term prognosis [2-4].

In addition to efforts being made to prevent AKI, research into early recovery and maximizing and maintaining recovery have gained more interest lately. It has been well-demonstrated that recovery of kidney function within 48 to 72 hours is associated with better outcomes as compared to individuals with prolonged and persistent AKI [5]. Studies have used different criteria to define kidney recovery. Traditionally, kidney recovery is defined as independence from kidney replacement therapy for up to 14 days in patients needing dialysis after an AKI episode. However, there is no definitive consensus to define complete and partial kidney recovery, especially after AKI episodes in HSCT patients [1]. Studies defining kidney recovery as a return to baseline kidney function could be cumbersome, as the baseline function is not quite evident in all acute conditions [6]. In a study by Pannu and colleagues, kidney recovery was defined as post-AKI serum creatinine level within 25% of baseline and independence from kidney replacement therapy [7]. In general, complete recovery can be defined as the resolution of all AKI criteria and partial recovery as the reduction in AKI stage considering clinical variables, including serum creatinine, glomerular filtration rate (GFR), and urine output [8].

It has been proposed that AKI and CKD are often a continuum. The Acute Kidney Disease and Renal Recovery (Acute Disease Quality Initiative, ADQI-16) workgroups have proposed multiple hypothetical AKI trajectories and shed light on kidney recovery. They have sub-classified AKI based on the timing of kidney recovery into 1) Transient AKI: which resolves in <48 hours, 2) Persistent AKI: if kidney dysfunction extends for >48 hours, 3) Acute kidney disease defined (AKD): as persistence of kidney injury from day 7 to day 90 after the initiating event. After 90 days, kidney failure is considered CKD. AKD represents the time window wherein critical interventions might be initiated to facilitate kidney recovery and mitigate the risk/severity of CKD.

The incidence of AKI and severe AKI (AKI Stage III) in patients undergoing hematopoietic stem cell transplant (HSCT) is approximately 55.1% and 8.3%, respectively [9]. Survivors of AKI after HSCT may have an increased risk of morbidity and mortality, especially if kidney replacement therapy (KRT) is needed. AKI after HSCT is attributed to multiple common risk factors, including allogeneic vs autologous transplant, and type and intensity of preconditioning regimen and post-transplant complications [10]. There is a paucity of literature on post-AKI kidney recovery in patients who underwent HSCT. Hence, we conducted a meta-analysis of kidney recovery in HSCT patients.

## Materials and methods

Search strategy

The Preferred Reporting Items for Systematic Reviews and Meta-Analysis (PRISMA) statement [11] was followed for conducting this systematic review. The Ovid MEDLINE, EMBASE, and Cochrane databases were systemically searched from their inception through August 2019. To identify the rates of kidney recovery from AKI, we conducted a literature search to identify all potential studies of patients who developed AKI after HSCT. Two investigators (S.K. and K.K.) performed an independent literature search using the search terms ((“bone marrow” OR “stem cell”) AND (“transplant” OR “transplantation”)) AND (“acute kidney injury” OR “acute renal failure” OR “renal replacement therapy”). Language restriction was not applied. Potentially related studies were manually reviewed using the references.

Study selection

Observational studies and clinical trials that provided data on the rate of kidney recovery in adult patients who developed AKI after HSCT were included in the meta-analysis. Two investigators (S.K. and K.K.) independently reviewed retrieved articles for their eligibility. A third investigator (W.C.) resolved the disagreement by moderating a consensus. Definitions of kidney recovery from AKI are shown in the table in the Appendix.

Data collection 

The information points that were collected from individual studies included article title, author names, year of the study, publication year, country where the study was conducted, patient characteristics, indication for HSCT, the definition of recovery of kidney function, the rates of kidney recovery from AKI or severe AKI requiring KRT, and extent of kidney recovery (complete vs. partial).

Statistical analysis

Adjusted point estimates of included studies were incorporated by the generic inverse variance method of DerSimonian-Laird, which assigned the weight of an individual study based on its variance. Due to the probability of between-study variance, we applied a random-effects model to pool the outcomes of interest, including the rate of kidney recovery. Cochran’s Q test (p<0.05 for a statistical significance) and I2 statistic (≤25% represents insignificant heterogeneity, 26% to 50% represents low heterogeneity, 51% to 75% represents moderate heterogeneity, and ≥75% represents high heterogeneity) were used to assess statistical heterogeneity. Publication bias was analyzed by the funnel plot and the Egger test 2. Meta-analysis was performed using Comprehensive Meta-Analysis software version 3.3.070 (Biostat Inc, New Jersey).

## Results

**Figure 1:**
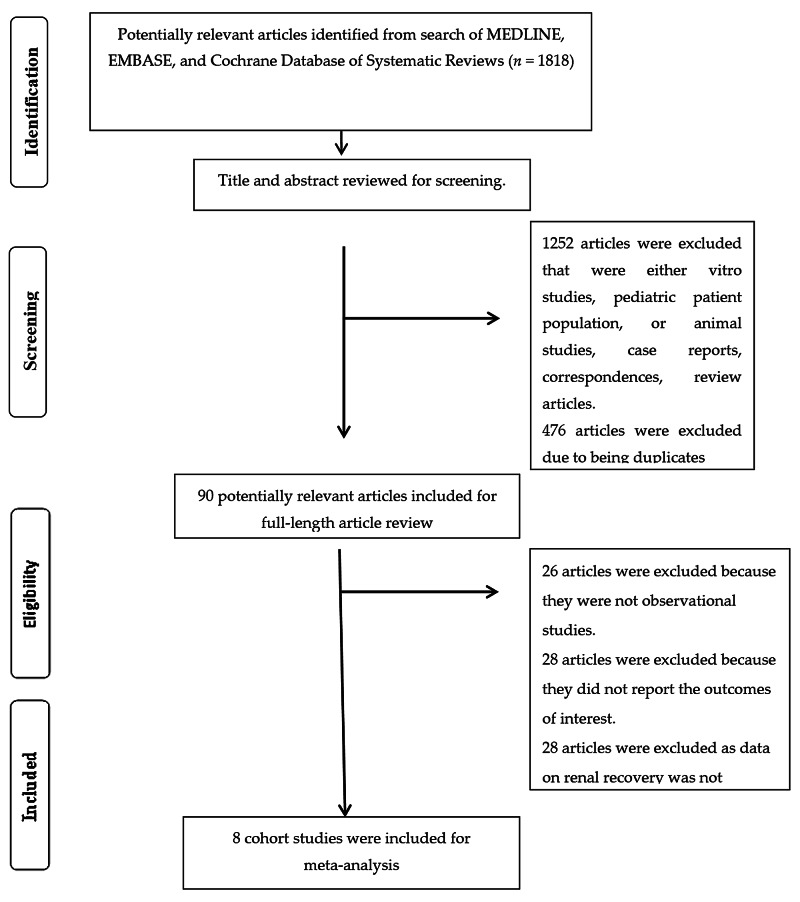
Outlines the identification of papers for inclusion

The initial search yielded a total of 1,818 articles for screening. Four-hundred seventy-eight duplicates were removed, and 1,262 articles were excluded for being in-vitro or animal investigation, including pediatric patients, case reports, correspondences, and review articles. Full-length reviews of 90 studies were performed. Twenty-eight studies were excluded due to not providing the outcome of interest, and 26 articles were not observational studies. The remaining 36 cohort studies with available data of AKI among patients undergoing HSCT were screened, and 28 studies were subsequently excluded, given no data of AKI recovery. Thus, eight cohort studies [12-19] with a total of 458 patients with AKI after HSCT with data of AKI recovery were finally enrolled as mentioned in Figure 1.

Rates of kidney recovery from acute kidney injury after HSCT

**Figure 2:**
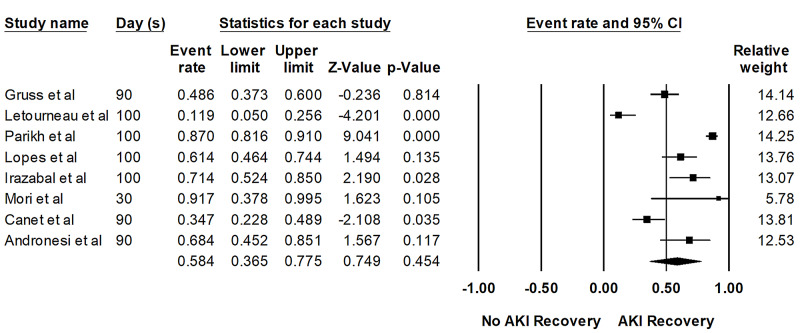
Forest plots of the involved studies assessing renal recovery rates from AKI after HSCT AKI: acute kidney injury; HSCT: hematopoietic stem cell transplant

**Figure 3:**
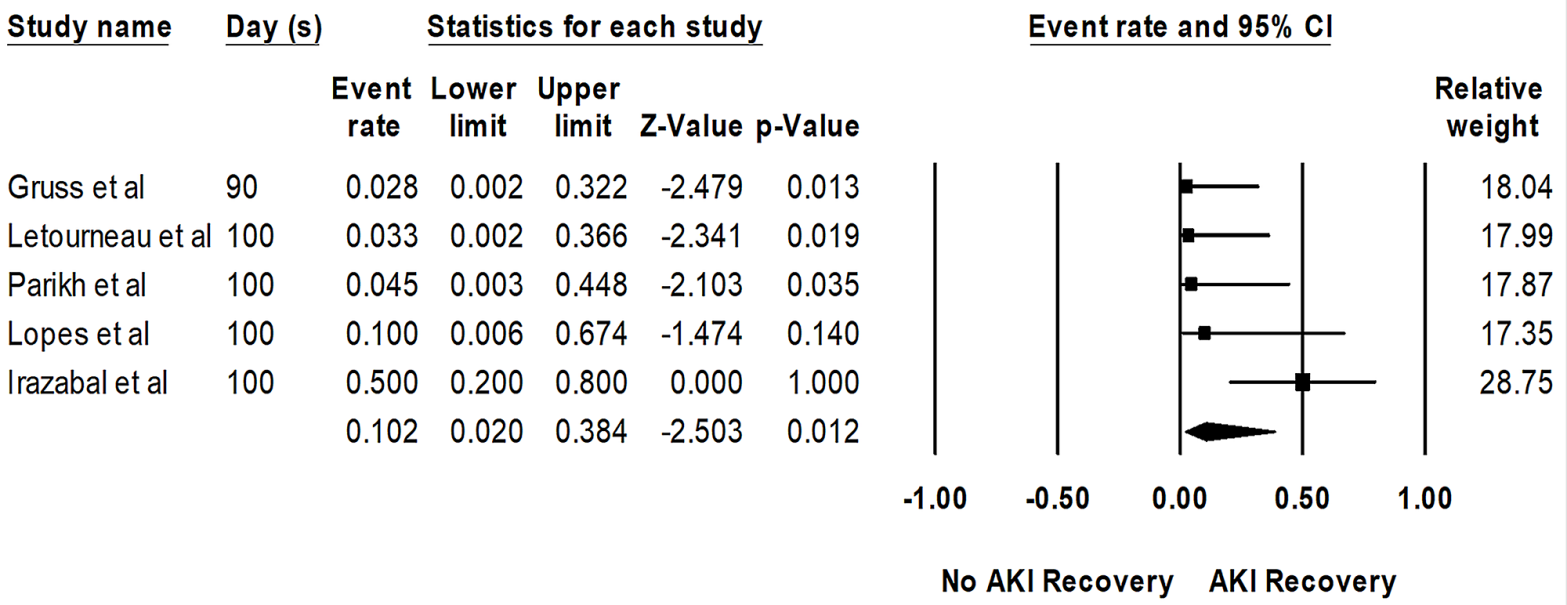
Forest plots of the involved studies assessing renal recovery rates from severe AKI requiring RRT after HSCT AKI: acute kidney injury; HSCT: hematopoietic stem cell transplant; RRT: renal replacement therapy

Overall, the pooled estimated rates of AKI recovery in patients with AKI and severe AKI requiring KRT within 100 days were 58% (95%CI: 37%-78%, I2 = 92%, Figure 2) and 10% (95%CI: 2%-4%, I2 = 58%, Figure 3), respectively.

Among patients with AKI recovery, the pooled estimated rates of complete AKI recovery and partial AKI recovery were 60% (95%CI: 39%-78%, I2 = 59%) and 29% (95%CI: 10%-61%, I2 = 85%), respectively. Sensitivity analyses were performed, after excluding a study by Mori et al. [16], given the limited number of patients with AKI in the study (N = 5). The pooled estimated rates of complete AKI recovery and partial AKI recovery from sensitivity analyses were 65% (95%CI: 48%-78%, I2 = 47%) and 20% (95%CI: 6%-50%, I2 = 87%), respectively.

**Figure 4:**
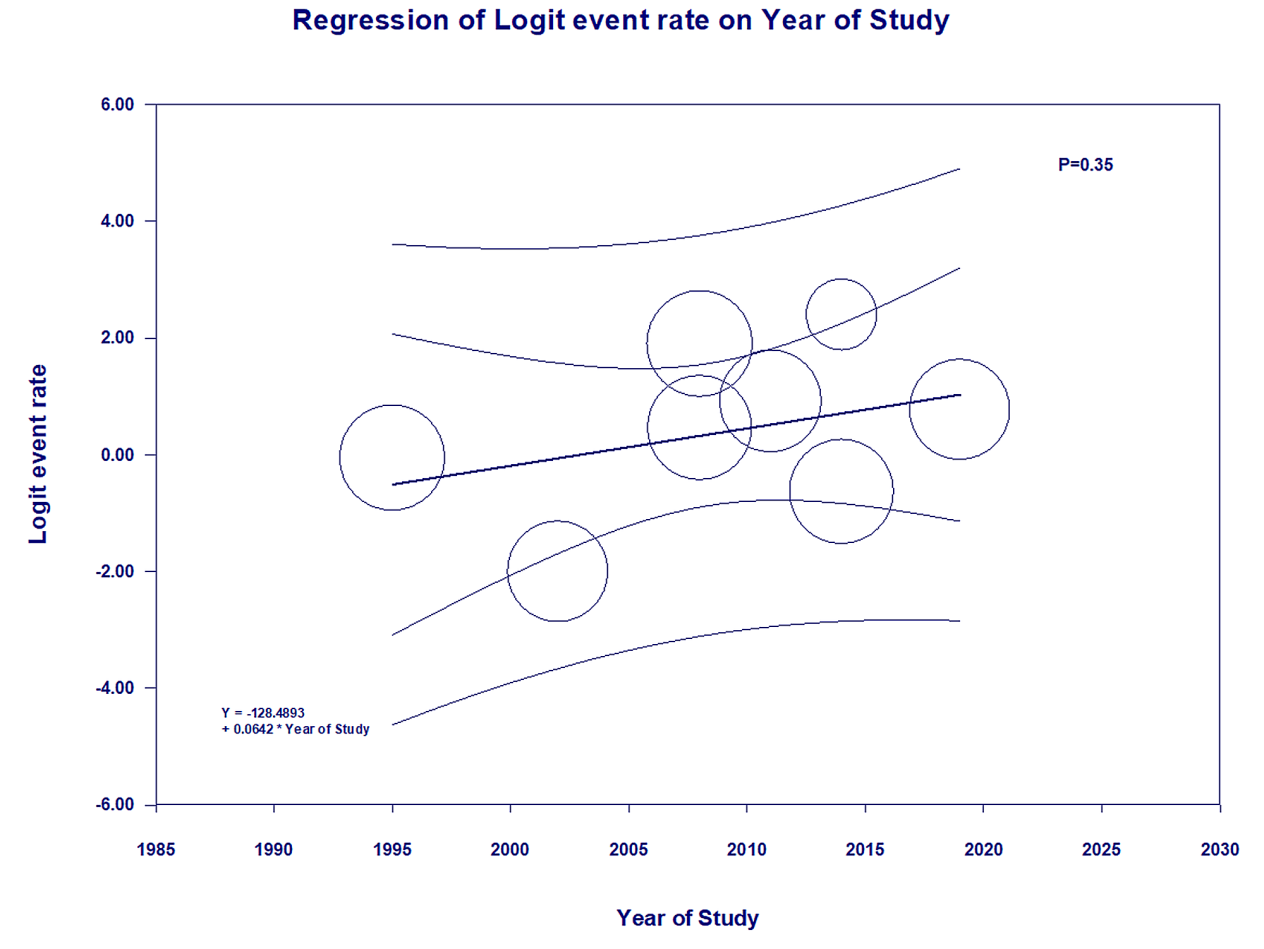
Meta-regression analysis showed no significant correlation between the year of the study and the rate of AKI recovery (p = 0.35) AKI: acute kidney injury

Although there was a trend with time toward higher rates of kidney recovery from AKI after HSCT, meta-regression showed no significant correlation between study year and the rate of AKI recovery (p = 0.35; Figure 4).

Evaluation for publication bias

**Figure 5:**
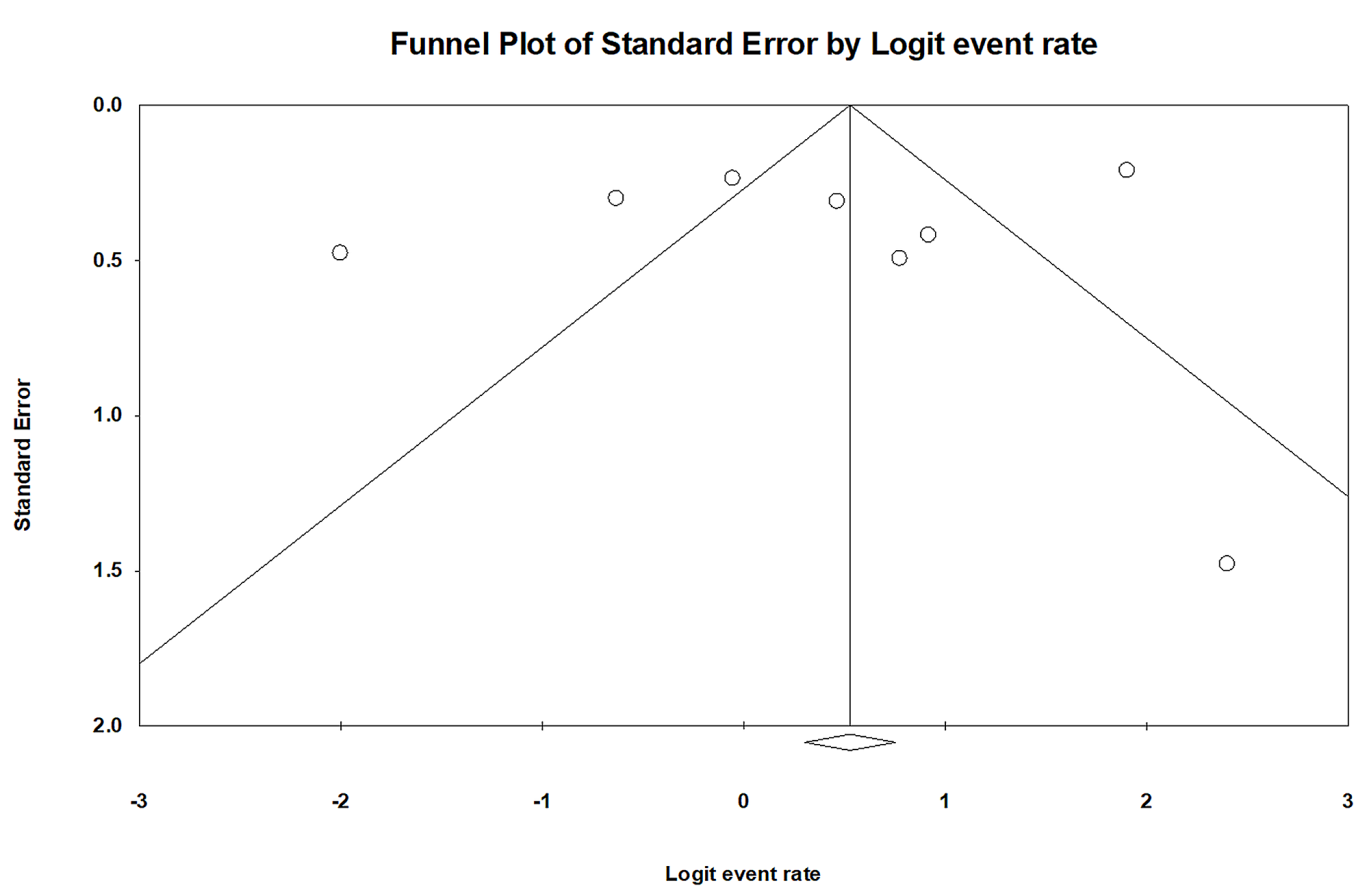
Funnel plot evaluating for publication bias evaluating the rate of AKI recovery AKI: acute kidney injury

The funnel plot (Figure 5) and Egger’s regression asymmetry tests were performed to assess publication bias in analysis evaluating the rate of AKI recovery. No significant publication bias in the meta-analysis evaluating the rates of AKI recovery among patients with AKI (p =0.59) was evident.

## Discussion

In the current systematic review, we assessed the rates of kidney function recovery among patients with AKI and severe AKI requiring renal replacement therapy (RRT) after HSCT. Among patients who had kidney recovery, complete recovery is achieved in two-thirds and partial recovery in about one-third. There was inconsistency in defining kidney recovery among the included studies. Complete kidney recovery has been defined as a return of serum creatinine to baseline by all studies except Parikh et al. [14], who defined complete recovery as the return of serum creatinine to less than 1.2 times baseline. In accord with the notion that allogeneic HSCT is associated with an increased incidence of AKI as compared to autologous, there was a signal from two studies [16,19] that autologous HSCT could have better recovery rates as compared to allogeneic HSCT, but more data are required to confirm these findings. Unfortunately, kidney recovery in those cases needing RRT was dismal except for the study by Irazabal et al. [16] in which half the patients recovered their kidney function after RRT. This is probably due to liberal RRT initiation criteria and the difference in illness severity. It is unclear if long-term follow-up for more than three months would generate more encouraging results.

Eight cohort studies with a total of 458 patients were included in our final analyses. A study by Gruss et al. [12] is perhaps the oldest. They reported an AKI incidence of only 26%, and the lower incidence could be secondary to a lesser number of allogenic transplants in their cohort. Elderly patients had a significant AKI incidence, with more recovery seen in the younger population. Seventeen patients underwent hemodialysis and mortality rates were remarkably high in RRT patients. The study by Létourneau et al. analyzed patients in the intensive care unit after HSCT [13]. They reported that low serum albumin, liver failure, and higher acute physiology and chronic health evaluation (APACHE) II scores were associated with higher AKI rates. Out of 14 patients who required RRT, no difference in outcome was reported based on the modality (continuous venovenous hemodiafiltration (CVVHDF) vs. intermittent hemodialysis (iHD)) of RRT. Parikh et al. retrospectively analyzed the long-term impact of AKI on survival in 200 patients with non-myeloablative HSCT [14]. They reported that about 13% of patients with persistently elevated creatinine at day 100 and patients who experienced AKI even after their recovery are at risk of developing hypertension, CKD, and increased mortality. Lopez et al. retrospectively studied the incidence of AKI and their long-term impact on outcomes after five years [15]. They reported that survival in patients after an AKI episode was linked to its severity (no AKI, 67.1%; RISK, 55.6%; injury plus failure, 33.3%). Similar results were reproduced in the study by Canet et al. where AKI stage 3 was corresponding with poor survival (19%) [18]. In a study by Irazabel et al., the rate of kidney recovery was delineated in 72% of patients, including 50 % of patients on dialysis [16]. The improved rates of recovery could be associated with the baseline characteristics of the patients, autologous stem cell transplant, or methylprednisone used for the treatment of engraftment syndrome. However, the incidence of RRT did not differ in patients with or without steroids. In the retrospective study by Mori et al. [17], 67 patients who received tacrolimus and teicoplanin concomitantly after allogeneic HSCT were evaluated, the rise in serum creatinine was reversible, and none in the study required RRT. In the prospective study by Andronesi et al. that reported 185 patients with underlying multiple myeloma after HSCT, only 10% had AKI and two-thirds of those patients had complete recovery at three months [19]. The improved outcomes could be attributed to underlying multiple myeloma and autologous stem cell transplant.

The pathophysiology of kidney recovery is complex, including the interplay between several factors and is still being explored. The reparative capacity of proximal tubular epithelial cells ultimately translates to changes in functional kidney reserve [20-21]. With transient AKI episodes, multiple compensatory mechanisms contribute to minimize the acceleration of kidney damage or promote tubular cell repair. However, with prolonged and sustained inflammatory or hypoxic episodes, there could be oxidative stress with maladaptive cell cycle arrest, which further contributes to the activation of additional inflammatory and profibrotic factors [22]. The inflammatory and endothelial cells eventually transform to profibrotic cells and fibroblasts [23], ultimately leading to tubulointerstitial fibrosis [24-25]. Given the challenges of serum creatinine in estimating kidney functions, there is a great utility of biomarkers to guide the early identification of AKI, potentially leading to early intervention and recovery.

Kidney recovery after AKI has gained more emphasis due to its importance for better clinical outcomes [26]. The American Society of Nephrology and acute disease quality initiative (ADQI) provide care bundles related to post-AKI care to further prevent progression to acute kidney disease [27]. In an observational cohort study by Harel et al., post-AKI patients who received follow-up in nephrology outpatient clinics demonstrated improved overall survival [28]. A study by Siew et al. illustrated that only 8.5% of AKI survivors were referred to nephrologists in the first year for follow-up and have a sustained high mortality rate [29]. Kidney Disease Improving Global Outcomes (KDIGO) guidelines recommended at least three months follow-up after an AKI episode. Specific consensus on frequency and timing of nephrology follow-up post-AKI in HSCT patients is lacking.

Implementation of a post-AKI care bundle (KAMPS - Kidney function, Advocacy, Medications, Pressure, Sick day protocol) includes periodic assessment of kidney function, checking proteinuria/albuminuria at specific intervals, patient and guardian education regarding AKI, and CKD during hospitalizations and at the time of discharge. Post-AKI, documentation of acute kidney disease, emphasis on avoiding nephrotoxic medications with special attention to angiotensin-converting enzyme (ACE)/angiotensin II type 1 receptor blockers (ARBs), and medication reconciliation post-discharge are a few measures to be emphasized. Educating patients regarding blood pressure goals and targets, checking daily weights, and the use of diuretics should be performed periodically [27]. These general measures can be applied to post-HSCT patients to help prevent the risk of further progression.

Routine end-stage renal disease (ESRD) bundle cannot be completely applied to patients with AKD. In patients requiring kidney replacement therapy (KRT) after AKI, special attention to be focused on patients’ dry weight, volume status, and diuretics in maintaining residual kidney function. Patients should be counseled on dialysis modalities and the importance to avoid hypotension episodes during RRT. Assessment of kidney clearance by GFR, cystatin C, documentation of urine output, several weekly pre-dialysis creatinine measurements, 24-hour urine for blood urea nitrogen (BUN), and creatinine clearances can identify kidney recovery (WATCH-ME) [27].

Limitations of this report include that we used cohort studies in our systematic review. They might not identify any causal relationship between patients with kidney recovery rates and needing KRT [30]. In addition, data on the impact of AKI recovery on outcomes among HSCT patients were limited. In patients with complete and partial recovery, there is a paucity of data regarding parameters like time to recovery, length of stay, ventilator requirement, long-term outcomes, and cost in the included studies. The mortality rate of patients who recovered compared to patients with no kidney recovery could not be obtained. Lastly, measures to promote kidney recovery among included studies are limited.

## Conclusions

As evident from our review, kidney recovery from AKI after HSCT occurs in approximately 58% among patients with AKI and 10% among patients with AKI requiring KRT, respectively. AKI among patients undergoing HCT is associated with high mortality. We will need larger studies to evaluate whether greater rates of AKI recovery results in better patient survival. Given low recovery and high mortality in AKI patients requiring KRT, potential disease burden with partial recovery, we should continue to increase awareness and pay more attention to their recovery phase to maximize and maintain kidney recovery. Future research and studies are required to streamline the optimal approach to enhance kidney recovery from AKI after HSCT.

## Appendices

**Table 1:** Major characteristic of studies involved in this meta-analysis of the rate of renal recovery and mortality among patients with hematopoietic stem cell transplantation Abbreviations: AKI- Acute Kidney Injury; AKIN- Acute Kidney Injury Network; KDIGO- Kidney Disease Improving Global Outcomes; RIFLE- Risk, Injury, Failure, Loss of Kidney Function, and End-Stage Kidney Disease; HSCT- Hematopoietic Cell Transplantation; HSCT- Hematopoietic Stem Cell Transplantation; RRT- Renal Replacement Therapy; GFR- Glomerular Filtration Rate; HD- Hemodialysis; AML- Acute Myeloid Leukemia; ALL- Acute Lymphocytic Leukemia; MM- Multiple Myeloma; HL- Hodgkin’s Lymphoma; NHL- Non-Hodgkin’s Lymphoma; USA- United States of America

Study	Year	Country	Patients	Indication for HSCT	AKI Definition	Number of patients with AKI	Rate of renal recovery (complete and partial)	Rate of renal recovery after RRT
Gruss et al. [22]	1995	Madrid, Spain	Autologous and allogeneic HSCT	Acute leukemia and lymphomas	Doubling of baseline serum creatinine	72	3-month renal total recovery = 35/72 = 49%	3-month recovery after RRT = 0/17 = 0%
Letourneau et al. [23]	2002	Canada	Autologous and Allogeneic HSCT	Hematological malignancies	Doubling of serum creatinine	42	100-day renal total recovery = 5/42= 12%, Complete recovery; 3/42=7.1%, Partial Recovery=2/42= 5%	100-day recovery after RRT = 0/14 = 0%
Parikh et al. [24]	2008	Seattle Washington, USA	Nonmyeloablative HSCT	Hematological malignancies	>2-fold increase in serum creatinine or requirement of dialysis.	200	100-day total renal recovery=174/200= 87%, Complete renal recovery (< 1.2 times baseline) = 100/200= 50%, Partial recovery (1.2 to 1.5 times baseline) = 74/200=37%	100-day recovery after RRT 0/10 = 0 %
Lopes et al. [25]	2008	Portugal	Reduced Intensity Conditioning	Hematological Malignancies	RIFLE	44	100-day total renal recovery = 27/44=61.3%, Complete renal recovery=21/44 = 48%, Partial renal recovery = 6/44 = 14%	100-day recovery after RRT = 0/4 = 0%
Irazabal et al. [26]	2011	Mayo, Rochester, USA	Autologous stem cell transplant	AL amyloidosis	AKIN	27	100-day total renal recovery = 20/28 = 72%	100-day recovery after RRT = 4/8= 50%
Mori et al. [27]	2014	Tokyo, Japan	Allogeneic Hematopoietic stem cell transplant	Hematological Malignancies	>50% rise in serum creatinine	5	30 day, partial recovery of renal function = 5/5 = 100%	None required RRT
Canet et al. [28]	2014	Paris, France	Allogenic Hematopoietic stem cell transplant	AML, ALL, Lymphoma	KDIGO	49	3 month total renal recovery = 17/49 = 35%	N/A
Andronesi et al. [29]	2019	Romania	Autologous stem cell transplant	Multiple myeloma	KDIGO	19	3 month total renal recovery (S Cr <0.3 mg/dl from baseline) = 13/19 = 68.4%	None required RRT

## References

[1] Forni LG, Darmon M, Ostermann M (2017). Renal recovery after acute kidney injury. Intensive Care Med.

[2] Kellum JA, Sileanu FE, Bihorac A, Hoste EA, Chawla LS (2017). Recovery after acute kidney injury. Am J Respir Crit Care Med.

[3] Thongprayoon C, Hansrivijit P, Kovvuru K (2020). Diagnostics, risk factors, treatment and outcomes of acute kidney injury in a new paradigm. J Clin Med.

[4] Thongprayoon C, Lertjitbanjong P, Cheungpasitporn W (2020). Incidence and impact of acute kidney injury on patients with implantable left ventricular assist devices: a meta-analysis. Ren Fail.

[5] Coca SG, King JT, Jr. Jr., Rosenthal RA, Perkal MF, Parikh CR (2010). The duration of postoperative acute kidney injury is an additional parameter predicting long-term survival in diabetic veterans. Kidney Int.

[6] Cerda J, Liu KD, Cruz DN (2015). Promoting kidney function recovery in patients with AKI requiring RRT. Clin J Am Soc Nephrol.

[7] Pannu N, James M, Hemmelgarn B, Klarenbach S, Alberta Kidney Disease Network (2013). Association between AKI, recovery of renal function, and long-term outcomes after hospital discharge. Clin J Am Soc Nephrol.

[8] Endre ZH (2018). Assessing renal recovery after acute kidney injury: can biomarkers help?. Nephron.

[9] Kanduri SR, Cheungpasitporn W, Thongprayoon C (2020). Incidence and mortality of acute kidney injury in patients undergoing hematopoietic stem cell transplantation: a systematic review and meta-analysis. QJM.

[10] Renaghan AD, Jaimes EA, Malyszko J, Perazella MA, Sprangers B, Rosner MH (2019). Acute kidney injury and CKD associated with hematopoietic stem cell transplantation. Clin J Am Soc Nephrol.

[11] Moher D, Liberati A, Tetzlaff J, Altman DG (2009). Preferred reporting items for systematic reviews and meta-analyses: the PRISMA statement. PLoS Med.

[12] Gruss E, Bernis C, Tomas JF (1995). Acute renal failure in patients following bone marrow transplantation: prevalence, risk factors and outcome. Am J Nephrol.

[13] Letourneau I, Dorval M, Belanger R, Legare M, Fortier L, Leblanc M (2002). Acute renal failure in bone marrow transplant patients admitted to the intensive care unit. Nephron.

[14] Parikh CR, Yarlagadda SG, Storer B, Sorror M, Storb R, Sandmaier B (2008). Impact of acute kidney injury on long-term mortality after nonmyeloablative hematopoietic cell transplantation. Biol Blood Marrow Transplant.

[15] Lopes JA, Gonçalves S, Jorge S (2008). Contemporary analysis of the influence of acute kidney injury after reduced intensity conditioning haematopoietic cell transplantation on long-term survival. Bone Marrow Transplant.

[16] Irazabal MV, Eirin A, Gertz MA (2012). Acute kidney injury during leukocyte engraftment after autologous stem cell transplantation in patients with light-chain amyloidosis. Am J Hematol.

[17] Mori T, Shimizu T, Kato J (2014). Nephrotoxicity of concomitant use of tacrolimus and teicoplanin in allogeneic hematopoietic stem cell transplant recipients. Transpl Infect Dis.

[18] Canet E, Lengline E, Zafrani L, Peraldi MN, Socie G, Azoulay E (2014). Acute kidney injury in critically ill allo-HSCT recipients. Bone Marrow Transplant.

[19] Andronesi AG, Tanase AD, Sorohan BM (2019). Incidence and risk factors for acute kidney injury following autologous stem cell transplantation for multiple myeloma. Cancer Med.

[20] Zuk A, Bonventre JV (2016). Acute kidney injury. Annu Rev Med.

[21] Ferenbach DA, Bonventre JV (2015). Mechanisms of maladaptive repair after AKI leading to accelerated kidney ageing and CKD. Nat Rev Nephrol.

[22] Venkatachalam MA, Weinberg JM, Kriz W, Bidani AK (2015). Failed tubule recovery, AKI-CKD transition, and kidney disease progression. J Am Soc Nephrol.

[23] Kumar S, Liu J, McMahon AP (2014). Defining the acute kidney injury and repair transcriptome. Semin Nephrol.

[24] Basile DP, Donohoe D, Roethe K, Osborn JL (2001). Renal ischemic injury results in permanent damage to peritubular capillaries and influences long-term function. Am J Physiol Renal Physiol.

[25] Basile DP (2004). Rarefaction of peritubular capillaries following ischemic acute renal failure: a potential factor predisposing to progressive nephropathy. Curr Opin Nephrol Hypertens.

[26] Thongprayoon C, Kaewput W, Kovvuru K (2020). Promises of big data and artificial intelligence in nephrology and transplantation. J Clin Med.

[27] Kashani K, Ding X, Pickkers P (2019). Quality improvement goals for acute kidney injury. Clin J Am Soc Nephrol.

[28] Harel Z, Wald R, Bargman JM (2013). Nephrologist follow-up improves all-cause mortality of severe acute kidney injury survivors. Kidney Int.

[29] Fortrie G, de Geus HRH, Betjes MGH (2019). The aftermath of acute kidney injury: a narrative review of long-term mortality and renal function. Crit Care.

[30] Kashani K, Cheungpasitporn W, Ronco C (2017). Biomarkers of acute kidney injury: the pathway from discovery to clinical adoption. Clin Chem Lab Med.

